# The Pathophysiological Mechanisms of Glia in Animal Models of Chronic Orofacial Pain: A Systematic Review

**DOI:** 10.3390/pathophysiology33030054

**Published:** 2026-07-21

**Authors:** Afonso Brito, Bruno Daniel Carneiro, Daniel Humberto Pozza

**Affiliations:** 1 Unit of Experimental Biology, Department of Biomedicine, Faculty of Medicine, University of Porto, 4200-319 Porto, Portugal; afonsomariagb6@gmail.com (A.B.); bcarneiro@med.up.pt (B.D.C.); 2 Rheumatology Service, Unidade Local de Saúde do Alto Minho, Hospital Conde de Bertiandos, 4990-078 Ponte de Lima, Portugal; 3 Institute for Research and Innovation in Health and IBMC (i3S), University of Porto, 4200-135 Porto, Portugal

**Keywords:** chronic orofacial pain, pain, glial cells, microglia, astrocytes, satellite glial cells, neuroinflammation, animal models, translational research

## Abstract

**Background/Objectives**: Chronic orofacial pain represents a heterogeneous group of disorders affecting the trigeminal system and remains difficult to treat due to its complex pathophysiology, which involves glial cells in the development and maintenance of persistent pain states. This systematic review aimed to synthesize evidence from animal studies investigating the pathophysiological role of glial cells in chronic orofacial pain. **Methods**: After registering on PROSPERO, a systematic search of the literature, based on PRISMA guidelines, was conducted to identify experimental animal studies evaluating glial involvement in orofacial pain models. After screening and eligibility assessment, 10 studies met the inclusion criteria. Data regarding experimental models, glial populations investigated, and pain-related behavioral outcomes were extracted and qualitatively synthesized. **Results**: Activation of satellite glial cells, microglia, and astrocytes was consistently associated with increased neuroinflammatory signaling and enhanced neuronal excitability within trigeminal pathways, demonstrating that both peripheral and central nervous tissues were involved. Several studies reported that pharmacological modulation of glial activity may reduce pain-related behaviors. **Conclusions**: Glial cells are key modulators of chronic orofacial pain through neuroimmune interactions that contribute to peripheral and central sensitization. Although these findings highlight promising therapeutic targets, further translational research is required to clarify their relevance for human pain conditions.

## 1. Introduction

Chronic orofacial pain constitutes a group of highly prevalent and debilitating conditions affecting the craniofacial region, including temporomandibular disorders, trigeminal neuropathic pain, persistent idiopathic facial pain, post-herpetic neuralgia, and cancer-related pain syndromes [1,2,3,4,5,6,7,8,9]. These disorders are associated with profound impairments in mastication, speech, sleep, and social functioning, and exhibit strong comorbidity such as anxiety, depression, and catastrophizing behaviors [4,5,6,7,8,9]. Epidemiological data indicate that orofacial pain disorders affect up to 30% of the general population at some point in life, with chronic forms representing a substantial burden to healthcare systems [10,11,12]. Despite this impact, available pharmacological and non-pharmacological treatments remain limited in efficacy, and many patients develop refractory pain, underscoring the need for improved mechanistic understanding and therapeutic innovation [13,14,15].

Historically, the pathophysiology of chronic pain has been conceptualized predominantly in neuronal terms, focusing on peripheral nociceptor sensitization, ectopic discharges, and maladaptive synaptic plasticity within central nociceptive circuits [16,17,18]. However, over the last two decades, this has been fundamentally revised with the recognition that glial cells actively participate in pain processing, contributing both to the initiation and maintenance of chronic pain states due to neuroplastic and glioplastic changes [19,20,21,22,23]. Glial populations include mainly microglia, astrocytes, oligodendrocytes, satellite glial cells (SGCs), and Schwann cells, each of which interacts dynamically with neurons and immune mediators within the central and peripheral nervous systems [19,22,24].

A defining feature of chronic pain is the development of neuroinflammation, a process largely orchestrated by glial cells. Following tissue injury or nerve damage, microglia rapidly adopt an activated phenotype characterized by upregulation of ionized calcium-binding adaptor molecule-1 (Iba-1), morphological hypertrophy, and enhanced production of cytokines such as tumor necrosis factor-α (TNF-α), interleukin-1β (IL-1β), and interleukin-6 (IL-6) [19,23,25,26,27,28,29]. This glial activation is closely coupled with central sensitization in the trigeminal brainstem sensory nuclear complex, where microglia and astrocytes undergo marked glioplastic changes. These changes sustain heightened neuronal excitability and nociceptive transmission by releasing inflammatory mediators that act on neuronal receptors and intracellular signaling pathways, facilitating phosphorylation of N-methyl-D-aspartate (NMDA) receptors, downregulation of inhibitory GABAergic transmission, and increased expression of voltage-gated sodium and calcium channels, thereby promoting and maintaining persistent pain states [16,23,28].

Astrocytes contribute to the persistence of pain through longer-lasting alterations in glutamate uptake, potassium buffering, and gliotransmitter release, which sustain hyperexcitability within nociceptive networks [20,30,31]. In parallel, astrocytic production of chemokines such as C-C Motif Chemokine Ligand 2 (CCL2) and C-X-C Motif Chemokine Ligand 1 (CXCL1) further recruits and activates microglia, establishing a self-perpetuating neuroimmune loop [30,31]. Oligodendrocytes and Schwann cells, traditionally viewed as purely myelinating cells, are now recognized to modulate axonal conduction and inflammatory signaling, influencing neuropathic pain phenotypes [32,33]. In the peripheral nervous system, SGCs surrounding sensory neurons in the dorsal root and trigeminal ganglia exhibit marked phenotypic changes following inflammation or nerve injury. These include upregulation of glial fibrillary acidic protein (GFAP), increased gap-junction coupling via connexin-43, and enhanced purinergic receptor expression (P2X7, P2Y12), facilitating paracrine signaling that amplifies neuronal excitability [34,35]. Such mechanisms have been implicated in the generation of ectopic activity and peripheral sensitization in both spinal and trigeminal nociceptive systems [36]. While glial contributions to spinal pain have been extensively investigated, the trigeminal system exhibits unique anatomical and functional features that may confer distinct glial–neuronal interactions. The trigeminal ganglion contains heterogeneous sensory neuron populations innervating craniofacial tissues, and projects centrally to the spinal trigeminal nucleus complex, which integrates nociceptive input from cutaneous, dental, and musculoskeletal structures [37,38,39].

Experimental models of orofacial pain, such as temporomandibular joint (TMJ) inflammation, masseter muscle injury, dental pulp inflammation, and infraorbital nerve ligation, consistently demonstrate robust activation of microglia and astrocytes in the spinal trigeminal nucleus and SGCs in the trigeminal ganglion [40,41]. In rodent models of TMJ inflammation induced by complete Freund’s adjuvant (CFA), increased GFAP expression in trigeminal ganglia SGCs and microglial activation in the medullary dorsal horn correlate with mechanical allodynia and facial grooming behaviors [40,42]. Infraorbital nerve injury models reveal sustained microglial activation and upregulation of purinergic and chemokine receptors, which are mechanistically linked to behavioral hypersensitivity [43]. Pharmacological or genetic inhibition of these glial pathways attenuates nocifensive responses, supporting a causal role for glial activation in pain maintenance [22,44].

At the molecular level, neuroglia communication involves a complex network of signaling pathways. ATP released from damaged neurons activates microglial P2X4 and P2X7 receptors, leading to p38 mitogen-activated protein kinase (MAPK) activation and brain-derived neurotrophic factor (BDNF) release, which in turn downregulates neuronal potassium-chloride cotransporter 2 (KCC2) and weakens inhibitory control [25,45,46]. Fractalkine (CX3CL1) signaling further sustains microglial activation, while astrocytic connexins and pannexins regulate intercellular propagation of calcium waves and inflammatory mediators [47,48,49]. Additionally, complement components (C1q, C3) and toll-like receptors (TLR2, TLR4) contribute to synaptic remodeling and neuroimmune activation in chronic pain states [50,51]. Beyond classical inflammatory cascades, emerging evidence implicates metabolic reprogramming, oxidative stress, and epigenetic regulation in glial-mediated pain chronification. Activated glia exhibit shifts towards glycolytic metabolism and increased mitochondrial reactive oxygen species (ROS) production, which further amplify inflammatory signaling [52,53,54]. Epigenetic modifications, including histone acetylation and DNA methylation in glial cells, modulate the expression of cytokines and ion channels involved in nociception [55,56]. These mechanisms may underlie long-lasting alterations in pain sensitivity and contribute to the resistance of chronic pain to conventional therapies.

Importantly, sex-dependent differences in glial responses have been described, with microglial signaling playing a dominant role in males and T-cell-mediated mechanisms being more prominent in females, suggesting that glial contributions to orofacial pain may also exhibit sexual dimorphism [57,58]. This highlights the importance of synthesizing animal data with attention to biological variables that influence translational relevance.

Despite extensive preclinical evidence, clinical translation of glia-targeted therapies has been limited. Agents such as minocycline, propentofylline, and ibudilast show robust antinociceptive effects in animal models but inconsistent efficacy in human trials [15,59,60]. This discrepancy underscores the need for a systematic appraisal of preclinical data, with careful consideration of experimental models, outcome measures, and mechanistic pathways relevant to orofacial pain. To date, although narrative reviews have addressed glial involvement in pain more broadly [19,20,21,22,30], a limited number of works have focused on orofacial pain [61]. No comprehensive systematic synthesis that has specifically examined how glial activation or modulation of glia-related molecular pathways influences outcomes in animal models of chronic orofacial pain was found. Such a synthesis is critical to identify convergent mechanisms, methodological limitations, and translational opportunities.

The present systematic review aimed to evaluate the available evidence from rodent models of chronic orofacial pain regarding the effects of glial activation and modulation of glia-related molecular pathways on pain-related behaviors and functional outcomes. By integrating molecular, cellular, and behavioral findings, this work seeks to clarify the mechanistic contribution of glia to orofacial pain chronification and to inform future therapeutic strategies targeting neuroimmune interactions within trigeminal nociceptive circuits.

## 2. Materials and Methods

A systematic review was undertaken following the Preferred Reported Items for Systematic Reviews and Meta-Analyses (PRISMA) guidelines [62,63]. The PRISMA 2020 Checklist is available as Supplementary Material Table S1.

### 2.1. PICO Question

The PICO question that served as the base for this work was as follows: “In animal models of chronic orofacial pain (P), what is the effect of glial activation or modulation of glia-related molecular pathways (I) compared with control or sham conditions (C) on pain intensity, hyperalgesia/allodynia, and functional impact (O)?”.

### 2.2. Eligibility Criteria

The inclusion criteria used were as follows: experimental in vivo studies—written in English with full-text PDF available for retrieval and analysis, conducted in rodent models (rats or mice) of chronic orofacial neuropathic and/or inflammatory pain induced through validated experimental procedures, and designed to induce or mimic chronic orofacial pain and/or to modulate glial activation or glia-related molecular signaling pathways (including morphological alterations, phenotypic changes, or intracellular signaling cascades)—and studies reporting at least one of the following outcomes: pain intensity, hyperalgesia and/or allodynia and/or functional impact (e.g., behavioral or affective alterations associated with pain).

The exclusion criteria used were as follows: experimental studies conducted exclusively in cell cultures, organotypic slices, or isolated tissues without an in vivo animal model of orofacial pain; studies primarily designed to evaluate the analgesic efficacy of a drug or compound in chronic pain models without investigating glial activation, glial modulation, or associated molecular signaling mechanisms; studies conducted in non-rodent species or in animal models not specifically related to chronic orofacial neuropathic or inflammatory pain; systematic reviews, narrative reviews, meta-analyses, scoping reviews, editorials, commentaries, letters to the editor, opinion papers, conference abstracts, dissertations, theses, preprints, technical reports, and other forms of gray literature; articles not published in English or without accessible full-text versions available for analysis (after request from the authors).

### 2.3. Search Strategy

The study protocol was registered in PROSPERO (International Prospective Register of Systematic Reviews) under the registration number CRD420251160245 in October 2025.

A systematic literature search was conducted in October 2025 in three electronic databases: PubMed, Web of Science (WoS), and Scopus. The search strategy combined controlled vocabulary (MeSH/keywords) and free-text terms, adapted to each database. For PubMed, the following MeSH-based query was used: (“Glial Cells”[MeSH Terms] OR “Astrocytes”[MeSH Terms] OR “Microglia”[MeSH Terms] OR “Oligodendroglia”[MeSH Terms]) AND (“Chronic Pain”[MeSH Terms] OR “Neuropathic Pain”[MeSH Terms] OR “Acute Pain”[MeSH Terms] OR “Neuroinflammation”[MeSH Terms]). For WoS, the topic-based query (“Neuroglia” AND “Chronic Orofacial Pain”) was applied. For Scopus, the query string TITLE-ABS-KEY (Neuroglia AND Chronic Orofacial Pain) was used. It is important to highlight that, although our focus was on orofacial pain, we opted to use this general query in order to avoid missing potentially relevant articles. Furthermore, there is no MeSH term for chronic orofacial pain. In March 2026, an additional manual search was performed utilizing the keywords: “orofacial pain”, “satellite glial cells”, “microglia” and “astrocytes” to identify any relevant articles missed during the initial search, and prior to manuscript finalization.

### 2.4. Manuscript Selection

Duplicate records were first identified and excluded prior to screening. Subsequently, two independent researchers conducted an initial assessment of titles and abstracts to determine study eligibility during October, November and December of 2025.

Manuscripts were selected in the Rayyan platform (https://www.rayyan.ai, accessed on 20 October 2025) with “blind mode” activated for the rigor of the process. Conflicts were resolved at a meeting in December 2025, with careful analysis and discussion to reach an agreement. Full-text articles were subsequently reviewed to determine eligibility for inclusion. Furthermore, the included manuscripts were carefully analyzed to ensure appropriate inclusion. A third researcher helped to resolve any doubts.

The studies were systematically assessed to include only those with suitable methodologies and relevant outcomes for the present manuscript, ensuring the results’ validity and reliability. A third researcher reviewed all the information. In cases of missing information or unavailable full texts, the corresponding authors were approached for clarification or access. The level of agreement was assessed using the Kappa test [64]. Additionally, in March 2026, the manual search for up-to-date literature, conducted during manuscript writing, resulted in one work identified as eligible for inclusion.

Eligible studies were reviewed, and data were manually extracted by two authors and compiled into a structured table for analysis in the months of January, February and March of 2026.

For each included study, we extracted the following data: first author, year of publication, country, study design, animal model selected and its characteristics (age, sex and weight), experimental intervention(s) and methodology for behavior assessment, molecular and cellular targets, and all predefined outcomes. Primary outcomes focused on pain-related behavioral measures, including changes in pain intensity and the development of hyperalgesia and/or allodynia, typically assessed through validated behavioral paradigms such as von Frey mechanical thresholds, operant conflict tests, and other nociceptive response assays. Secondary outcomes encompassed functional and affective-behavioral alterations associated with chronic orofacial pain, as well as morphological and molecular changes in glial cells and related signaling pathways (e.g., cytokine expression, purinergic signaling, complement activation, ion channel modulation), which were analyzed to explore mechanistic correlates of pain behavior.

### 2.5. Risk of Bias Assessment

The risk of bias for each included study was independently assessed by two authors using the SYRCLE’s risk of bias tool for animal studies [65], which evaluates bias across ten domains. Each domain is rated from “low” to “high” risk of bias, which informs the overall study-level judgment. Final assessments were visualized and summarized using an intuitive table.

### 2.6. Data Synthesis

Due to substantial heterogeneity in the experimental pain models, animal species and sex, intervention protocols, molecular targets, behavioral outcome measures, and timing of assessments, a quantitative meta-analysis was not considered appropriate. Therefore, the findings were synthesized qualitatively.

## 3. Results

Our initial search across all three databases yielded 617 articles: 193 from PubMed, 19 from WoS, and 405 from Scopus. After removal of 64 duplicate records, the remaining studies were screened based on title and abstract. Following this screening process, studies that failed to meet the predefined eligibility criteria were excluded.

A total of 12 reports were considered potentially eligible and underwent full-text assessment. Two studies were subsequently excluded: one due to the use of a non-orofacial pain model (sciatic nerve injury); one because the full-text version in English was unavailable.

**Figure 1:**
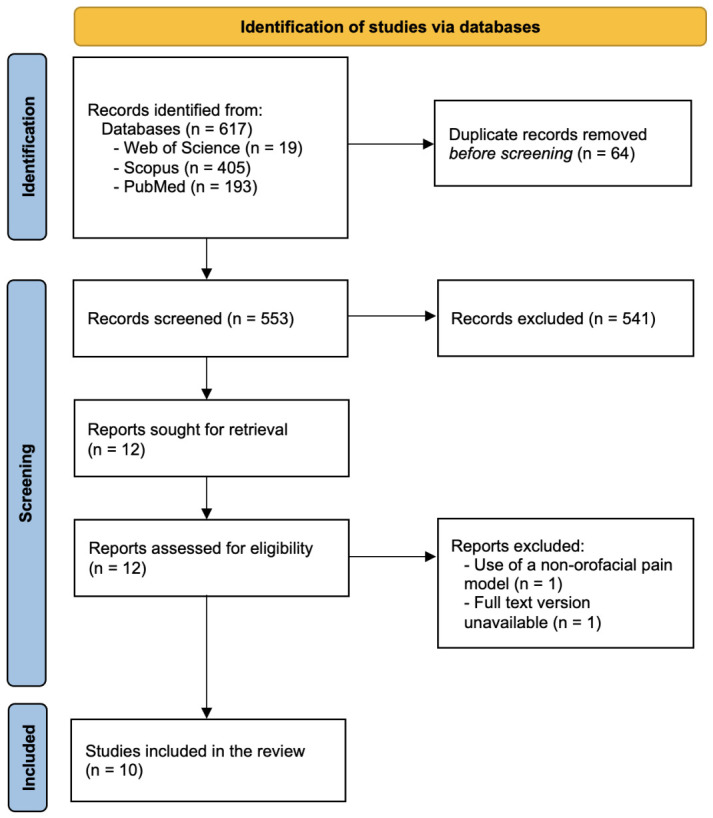
PRISMA flow diagram of study selection.

A final total of 10 studies met all inclusion criteria and were included in this systematic review. The flow diagram of the study selection process is presented in Figure 1. Inter-reviewer agreement during the screening process was high (Cohen’s kappa of 0.93). The additional manual search retrieved 138 articles; however, none met the eligibility criteria. These studies were utilized solely to provide an updated context for the literature review.

### 3.1. Description of Included Studies

The characteristics of the included studies, including species, experimental models of orofacial pain, glial targets, and main outcomes, are summarized in Table 1. All studies were conducted in vivo using rodent models, including rats and mice. The main methodological similarities, differences, and convergent findings are presented in Table 2.

Most studies employed established experimental models of chronic orofacial neuropathic or inflammatory pain, most commonly chronic constriction injury of the infraorbital nerve (CCI-IoN), subcutaneous injection of CFA, or models of TMJ inflammation. These paradigms reproduce key features of human chronic orofacial pain conditions, including trigeminal neuralgia and temporomandibular disorders.

Mechanical hypersensitivity was the most commonly evaluated behavioral outcome, typically assessed through von Frey filament testing applied to the vibrissae pad, submandibular region, or other trigeminal territories. Other studies additionally examined thermal sensitivity or affective-behavioral responses such as anxiety- or depression-like behavior through the usage of validated tools (e.g., open field, elevated plus maze, or tail suspension tests).

Across the included studies, the investigated molecular mechanisms focused primarily on glial activation and glia-related signaling pathways involving microglia, astrocytes, or SGCs. These mechanisms included inflammatory cytokine signaling, purinergic signaling, complement cascade activation, ion channel regulation, and ATP- or adenosine-mediated neuron-glia communication. The main experimental findings were therefore organized according to the predominant glial populations and molecular pathways implicated in chronic orofacial pain.

### 3.2. Glial Activation in Peripheral Trigeminal Structures

In an inflammatory model induced by complete Freund’s adjuvant injection into the whisker pad, Aczél and collaborators [68] demonstrated increased expression of the tachykinin precursor 4 (Tac4) gene encoding hemokinin-1 in both sensory neurons and SGCs of the trigeminal ganglion. This upregulation followed a temporal pattern that closely paralleled reductions in facial mechanonociceptive thresholds, suggesting that hemokinin-1 signaling contributes to peripheral sensitization during inflammatory trigeminal pain. Similarly, Kushnir [71] showed that peripheral inflammation and infraorbital nerve axotomy significantly increased the responsiveness of SGCs to adenosine triphosphate (ATP) in trigeminal ganglia. Calcium imaging experiments revealed enhanced sensitivity mediated primarily through upregulated purinergic 2X (P2X) receptors, particularly the P2X2 and P2X5 subtypes. Pharmacological inhibition with P2X receptor antagonists markedly reduced ATP-evoked responses in SGCs from inflamed animals, indicating that inflammation promotes a functional shift from P2Y receptors- toward P2X receptors-mediated signaling. Additional evidence supporting the role of SGCs in trigeminal sensitization was provided by Vit and collaborators [73], who selectively silenced the potassium inwardly rectifying channel 4.1 (Kir4.1) in SGCs within the trigeminal ganglion. This manipulation induced pronounced pain-like behaviors in rats, including mechanical hypersensitivity, increased spontaneous eye-closure frequency, and altered performance in operant conflict paradigms, even in the absence of nerve injury.

These findings indicate that dysfunction of glial potassium buffering mechanisms may contribute to neuronal hyperexcitability and pain generation. Together, these studies highlight the critical contribution of SGCs and peripheral glial signaling within the trigeminal ganglion to the development of peripheral sensitization in chronic orofacial pain.

### 3.3. Central Glial Activation in Trigeminal Pain Pathways

Multiple studies reported marked activation of glial populations in central nociceptive structures following induction of orofacial pain. Using a model of TMJ inflammation, Villa [40] demonstrated significant activation of SGCs and resident macrophages in the trigeminal ganglion, accompanied by microglial activation in central regions including the trigeminal subnucleus caudalis and cervical dorsal horn. Morphological changes characteristic of activated microglia were observed, including enlarged cell bodies and shortened processes, indicating a reactive microglial phenotype. Notably, astrocytic activation was not observed during the early inflammatory phase, suggesting a temporally distinct contribution of different glial populations during the progression of trigeminal pain. Consistent with these findings, Lee and collaborators [72] reported distinct temporal patterns of microglial and astrocytic activation in the trigeminal nuclear complex following peripheral nerve injury. Microglial proliferation and morphological activation were detected early after mental nerve ligation, whereas astrocytic hypertrophy appeared later. This sequential activation suggests that microglia may initiate neuroinflammatory processes that are subsequently maintained or amplified by astrocytes.

Further evidence of central glial involvement was reported by Demartini [66], who investigated the role of transient receptor potential ankyrin 1 (TRPA1) channels in a chronic constriction injury model of the infraorbital nerve. Nerve injury induced increased expression of pro-inflammatory cytokines, including tumor necrosis factor alpha (TNF-α), interleukin (IL)-1β, and IL-6, together with reduced levels of anti-inflammatory cytokines such as IL-10 and IL-4. These changes were accompanied by increased activation of microglia and astrocytes across several regions of the trigeminal nociceptive pathway, including the trigeminal ganglia, trigeminal nucleus caudalis, medulla, and cervical spinal cord. Pharmacological blockade of TRPA1 significantly attenuated these inflammatory responses and reduced glial activation, suggesting that TRPA1 signaling participates in glia-mediated neuroinflammatory processes in trigeminal neuropathic pain.

Collectively, these findings indicate that both peripheral and central glial populations contribute to the pathophysiology of chronic orofacial pain through coordinated neuroinflammatory mechanisms.

### 3.4. Astrocyte-Mediated Signaling and Neuron–Glia Crosstalk

Several studies specifically highlighted the role of astrocytes in the neuron–glia interactions in the modulation of chronic orofacial pain. Lv and collaborators [69] investigated astrocyte-derived purinergic signaling in the ventral hippocampus using a chronic constriction injury model of the infraorbital nerve. The nerve injury induced pronounced mechanical allodynia accompanied by increased astrocytic activation and elevated extracellular adenosine levels within the hippocampal cornu ammonis area 1 (CA1). Microglial activation and increased expression of IL-17A were also observed, indicating a complex interaction between glial populations. Experimental inhibition of astrocytes or blockade of adenosine signaling pathways significantly reduced antidepressive-like behaviors associated with chronic trigeminal neuropathic pain, although mechanical hypersensitivity remained unchanged. These results suggest that astrocyte-derived adenosine signaling in limbic brain regions contributes primarily to affective components of chronic pain. Complementary findings were reported by Feng [67], who examined glial–neuron interactions in the medial prefrontal cortex (PFC) in a model of TMJ osteoarthritis induced by unilateral anterior crossbite. The authors demonstrated increased expression of the inflammatory cytokine IL-1β, predominantly localized in microglial cells, together with increased neuronal expression of secretory phospholipase A2 group III (PLA2-III). Pharmacological inhibition of IL-1β or genetic silencing of PLA2-III significantly reduced both orofacial mechanical hypersensitivity and widespread somatic hyperalgesia. These results indicate that microglia-derived cytokines may modulate neuronal signaling pathways that contribute to central sensitization and widespread pain.

These findings emphasize the importance of neuron–glia communication in the central nervous system as a key mechanism underlying the amplification and persistence of chronic orofacial pain.

### 3.5. Complement-Mediated Microglial Synaptic Remodeling

In 2025, Huang [74] investigated complement-dependent synaptic pruning in the rostral ventromedial medulla (RVM) in a rat model of TMJ inflammation. The authors observed increased expression of complement proteins C1q and C3 together with elevated microglial markers, including ionized calcium-binding adaptor molecule 1 (IBA1) and cluster of differentiation 68 (CD68). These changes were temporally correlated with the development of mechanical hypersensitivity. Further analyses revealed that microglial cells selectively targeted excitatory presynaptic terminals for phagocytosis through the complement C1q/C3-CR3 signaling pathway. Pharmacological inhibition of complement activation with C1q inhibitor or suppression of microglial activation with minocycline significantly reduced mechanical allodynia and attenuated microglial activation in the RVM.

These findings suggest that complement-mediated microglial synaptic pruning may contribute to maladaptive synaptic plasticity within descending pain modulatory circuits, thereby promoting the persistence of chronic orofacial pain.

### 3.6. Summary of Experimental Findings

**Figure 2:**
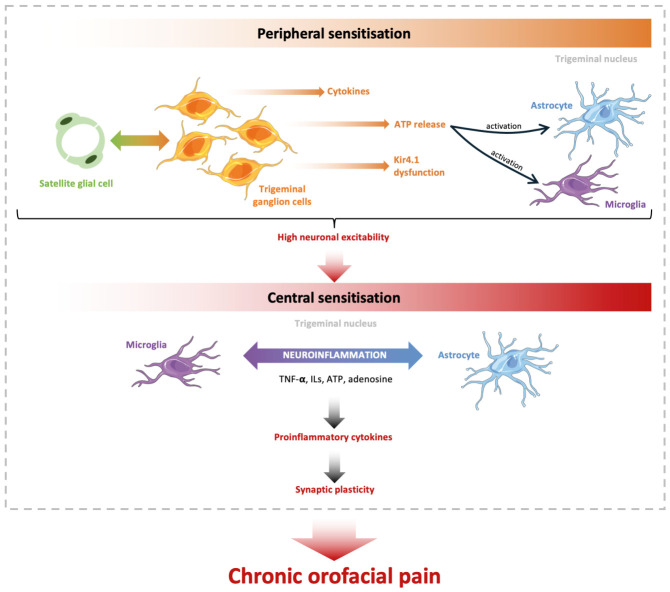
Neuron–glia interactions in chronic orofacial pain. ATP, adenosine triphosphate. Parts of this figure were drawn using pictures from Servier Medical Art by Servier (https://smart.servier.com), which is licensed under Attribution 4.0 International (Creative Commons CC BY 4.0); and finalized utilizing Microsoft PowerPoint software version 16.111 (Microsoft Corporation).

The investigated models of orofacial pathology demonstrated a clear neuroinflammatory cascade transitioning from peripheral injury to central sensitization within the trigeminal system. At the peripheral and ganglionic levels, localized insults—such as infraorbital or mental nerve injury and TMJ inflammation—upregulated neuronal Tac4, activated TRPA1 receptors, and disrupted satellite glial cell (SGC) homeostasis via the loss of Kir4.1 potassium channels and a shift toward purinergic P2X receptor dominance. As these amplified signals propagated into the central nervous system, they triggered a sequential glial response within the trigeminal nuclei, marked by early microglial activation followed by delayed, persistent astrocytic reinforcement. Activated microglia drove central sensitization by releasing pro-inflammatory cytokines like IL-1β and executing complement-mediated synaptic pruning (C1q/C3-CR3) in the rostral ventromedial medulla (RVM), while astrocytic pannexin-1 (Panx1)-mediated ATP release and sustained astrocyte-microglia crosstalk locked the system into a hyper-excitable state. Ultimately, this neuroimmune signaling cascaded beyond brainstem relays into the medial prefrontal cortex and ventral hippocampus, modulating both persistent mechanical hypersensitivity and the affective, anxio-depressive comorbidities of chronic orofacial pain (Figure 2).

**Figure 3:**
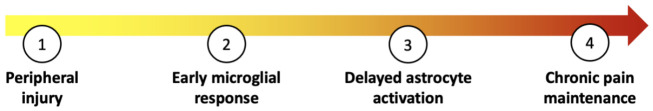
Temporal and spatial progression of glial activation during chronic orofacial pain.

These findings suggest a coordinated temporal sequence in which peripheral satellite glial cell activation precedes early microglial activation and subsequent astrocyte-mediated maintenance of chronic neuroinflammation, reinforcing the transition from peripheral to central sensitization (Figure 3).

Importantly, pharmacological or genetic interventions targeting glial signaling pathways frequently attenuated pain behaviors and inflammatory responses, supporting the hypothesis that glial cells represent key modulators of chronic orofacial pain.

### 3.7. Risk of Bias

**Figure 4:**
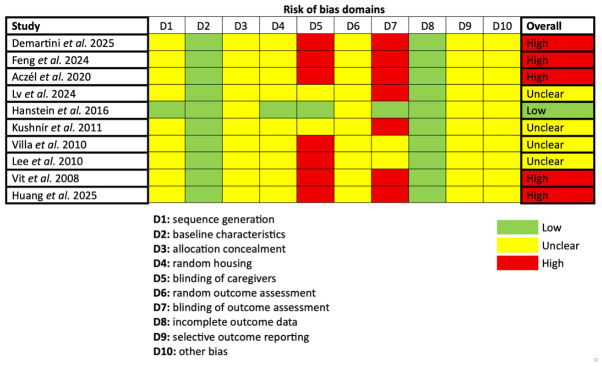
Risk of bias domain assessment of the 10 articles included in the review [40,66,67,68,69,70,71,72,73,74].

One study was assessed for bias, with an overall risk of bias rated as “low” [70]. Four studies were classified as having an “unclear” risk of bias [40,69,71,72]. Finally, five studies were classified with a “high” risk of bias [66,67,68,73,74]. The detailed information is shown in Figure 4.

## 4. Discussion

Across the compiled literature, convergent findings demonstrate that the activation and functional modulation of distinct glial populations constitute pivotal mechanisms driving the initiation and maintenance of persistent nociceptive states within trigeminal pathways. Although experimental paradigms varied across studies, a consistent pathophysiological pattern emerged: peripheral trigeminal injury or inflammation triggers robust neuroimmune cascades spanning both peripheral ganglionic structures and central nociceptive circuits, ultimately driving behavioral hypersensitivity and comorbid affective-related pain disturbances.

Although the included studies consistently demonstrated the involvement of glial cells in chronic orofacial pain mechanisms, they exhibited considerable methodological heterogeneity. Different experimental paradigms, including inflammatory and neuropathic models, target distinct aspects of trigeminal pathophysiology and therefore reproduce complementary rather than identical mechanisms. This diversity, together with variations in animal species, sex, molecular targets, and outcome measures, precluded direct quantitative comparison across studies. Nevertheless, all included models investigated neuroimmune processes within the trigeminal system, supporting their inclusion in the present qualitative synthesis. Furthermore, because only a limited number of studies included female animals [67,69,70,71,74], potential sex-related differences in glial responses should be considered when interpreting the translational relevance of the findings.

The included studies differed in terms of pain models, animal characteristics, behavioral assessments, molecular targets, and anatomical sites; however, they consistently demonstrated the involvement of glial cells in chronic orofacial pain. Neuropathic and inflammatory models differed in their initial mechanisms, but both converged toward enhanced neuroinflammation, increased neuronal excitability, and persistent pain [75,76]. These methodological differences should be considered when interpreting the findings but do not change the overall conclusion that glial activation plays a central role in chronic orofacial pain.

Beyond summarizing the findings of individual studies, the present systematic review identifies a coherent pathophysiological sequence underlying chronic orofacial pain. The available evidence consistently supports a model in which peripheral injury initially activates SGCs within the trigeminal ganglion, promoting neuronal hyperexcitability through purinergic signaling, cytokine release, and altered potassium homeostasis. These peripheral neuroimmune changes are followed by early activation of microglia within central trigeminal pathways, which amplify neuroinflammation through pro-inflammatory cytokines and complement signaling. Subsequently, astrocytes become activated and contribute to the maintenance rather than the initiation of persistent pain by sustaining maladaptive synaptic plasticity and prolonged neuroimmune communication. This temporal and spatial progression provides an integrated mechanistic framework that extends beyond the conclusions of the individual experimental studies.

The role of SGCs within the trigeminal ganglion as primary mediators of peripheral sensitization has been demonstrated [34,35]. Experimental manipulations disrupting purinergic signaling, potassium buffering capacity, or neuropeptide expression within SGCs consistently elicit pronounced nociceptive phenotypes. Critically, these nociceptive behaviors manifest even in the absence of clear, direct neuronal injury, corroborating the paradigm shift that sensory ganglia function as dynamic neuroimmune centers rather than passive relay stations. Previous mechanistical studies demonstrate that SGCs form extensive functional syncytia coupled via gap junctions and regulated by paracrine signaling. This architecture enables the rapid propagation of pro-inflammatory cascades that directly modulate adjacent neuronal excitability [77]. In the context of chronic orofacial pain, injury-evoked peripheral inputs from trigeminal afferents trigger robust neuroimmune activation within the ganglion, driving distinct glioplastic alterations that amplify nociceptor excitability and lower mechanical and thermal activation thresholds [23]. Consequently, these localized ganglionic processes serve as critical drivers of peripheral sensitization in both neuropathic and inflammatory pain states.

Beyond peripheral structures, several studies included in this review demonstrated the existence of glial activation within central components of the trigeminal nociceptive pathway, particularly the spinal trigeminal nucleus, RVM, and higher-order limbic regions. The temporal sequence observed in some models, characterized by early microglial activation followed by delayed astrocytic responses, supports the concept that microglia may act as initial sensors of neuronal injury, subsequently orchestrating broader neuroinflammatory cascades involving astrocytes and other immune mediators [37,72].

Within the trigeminal brainstem sensory nuclear complex, microglia and astrocytes undergo activation that enhances synaptic transmission and prolongs nociceptive signaling. These glial–neuronal interactions can persist long after the initiating injury has resolved, signifying a transition from adaptive plasticity to maladaptive, self-sustaining sensitization within trigeminal nociceptive pathways. Together, these findings highlight SGC-mediated ganglionic sensitization as a critical upstream driver of both peripheral and central mechanisms that maintain persistent orofacial pain. Additionally, this pattern may reflect distinct functional roles of glial subtypes in the initiation versus maintenance of chronic pain [23,78].

Importantly, these mechanisms appear to exhibit distinctive characteristics within the trigeminal system. Unlike spinal pain pathways, the trigeminal network involves highly specialized interactions among SGCs in the trigeminal ganglion, brainstem nociceptive nuclei, and supraspinal regions involved in emotional pain processing [37,79]. These anatomical and functional features may explain the complex sensory and affective manifestations commonly observed in chronic orofacial pain disorders.

Although both inflammatory and neuropathic models demonstrated robust glial activation, important differences emerged. Inflammatory models were characterized predominantly by rapid cytokine release and activation of SGC following tissue injury [68,71], whereas neuropathic models showed more sustained microglial activation, complement-mediated synaptic remodeling, and persistent astrocyte–microglia communication [66,69,72]. Nevertheless, both model types converged toward enhanced neuronal excitability, central sensitization, and persistent pain, indicating that distinct initiating events ultimately activate common neuroimmune pathways.

Another important mechanistic insight emerging from the reviewed studies concerns the role of neuron–glia communication in shaping both sensory and affective dimensions of pain. Interesting, astrocyte-derived purinergic signaling and microglia-derived cytokine release were shown to influence neural activity in limbic structures such as the hippocampus and medial PFC. These findings align with the concept that chronic pain is not solely a sensory disorder but a complex brain state involving emotional, cognitive, and motivational components [69], even in animal models of orofacial pain-like behavior.

Microglial involvement in structural synaptic remodeling extends beyond complement-dependent pruning, integrating a broader repertoire of inflammatory and receptor-mediated mechanisms that converge on nociceptive circuit reorganization. While complement-mediated synapse elimination, such as CR3-dependent microglial engulfment of glutamatergic inputs in the PFC, can disrupt top–down prefrontal cognitive control, microglial remodeling within spinal and descending pathways often targets inhibitory networks, thereby driving central sensitization. This aligns with findings that complement proteins contribute to synaptic refinement in descending pain modulatory regions like the RVM. However, chronic pain synaptic remodeling is not solely reliant on complement pathways: reactivated microglia release potent pro-inflammatory cytokines (including IL-1β, IL-6, and TNF-α) and interface with infiltrating immune signals like IFN-γ. These factors profoundly alter synaptic integrity through diverse receptor systems, including IL-1R1, IL-6R, TNFR, CX3CR1, CSF1R, and CD200R. These neuroimmune interactions impair dendritic spine dynamics, alter neurotransmitter release, and destabilize the excitatory-inhibitory balance. IL-1β, for instance, phosphorylates NMDA receptors to enhance glutamatergic hyperexcitability, whereas TNF-α and IL-6 modulate both Hebbian and homeostatic plasticity, promoting pathological synapse loss and mitochondrial dysfunction. Moreover, chemokine signaling via CX3CL1/CX3CR1 and CSF1/CSF1R facilitates activity-dependent microglial pruning, altering synapse density in central circuits. Thus, the aberrant orchestration of multiple microglial pruning mechanisms, CR3-complement engagement, and cytokine-driven structural alterations collectively compromise the excitatory-inhibitory equilibrium within pain-processing networks, reinforcing central hypersensitivity and sustaining the transition from acute to persistent pain [27,28,74,80,81,82].

Beyond the primary studies included in the formal analysis, the complementary manual search provided valuable contextual insight regarding the influence of glial cells in chronic pain. Specifically, these broader findings highlight that chronic pain reflects a pathological brain state characterized by dysregulated neurotransmission, neuroimmune activation, and maladaptive plasticity across distributed networks rather than a purely sensory disorder. Neuroimaging studies reveal elevated TSPO binding and increased myo-inositol, markers of microglial and astrocytic activation, in cortical and limbic regions including the insula, ACC, thalamus, and prefrontal cortex, changes that correlate with pain intensity and affective burden. Additionally, functional MRI indicated that glia-driven neuroinflammatory signaling contributes to large-scale network reorganization implicated in pain rumination, emotional amplification, and impaired cognitive control. Together, these observations suggest that glial signaling plays a central role in the neural plasticity underlying pain chronification and its associated mood disturbances, linking peripheral injury and central sensitization to the emergence of a persistent, affectively loaded pain state [83,84].

From a translational perspective, the findings summarized in this review provide important information regarding the concept that chronic orofacial pain may represent, at least in part, a disorder of dysregulated neuroimmune signaling within the trigeminal system [23,85,86]. This conceptual shift underscores the potential of targeting glial and immune pathways to modulate aberrant nociceptive processing. Nevertheless, translating robust preclinical evidence into effective clinical therapies remains a considerable challenge.

Pharmacological agents aimed at suppressing glial activation, such as minocycline, propentofylline, and ibudilast, have repeatedly demonstrated antinociceptive and anti-inflammatory efficacy in rodent models, yet clinical trials in humans have yielded inconsistent or only modest benefits [87,88,89]. These discrepancies likely reflect differences in dosing, timing of intervention, pain chronicity, and the complexity of human neuroimmune phenotypes, highlighting the need for better stratification, biomarker-guided trials, and a deeper understanding of glial heterogeneity in chronic orofacial pain. Additionally, differences in species-specific immune responses, variability in experimental pain models, and the complexity of human chronic-pain conditions, which certainly involve psychosocial and environmental influences, cannot be captured in animal paradigms.

Most preclinical work still uses short, acute/subacute paradigms that track evoked hypersensitivity over days or weeks, whereas chronic orofacial pain evolves over months and years with progressive plasticity from peripheral trigeminal afferents to brainstem and cortical circuits, limiting generalization. In rodents, evoked responses and seldom-captured spontaneous/ongoing pain dominate the clinical phenotype, a gap especially relevant to trigeminal disorders where brainstem reflexes (e.g., trigemino-facial blink reflex) and descending PAG-RVM modulation shape symptom maintenance. Models also show low heterogeneity; nearly all animals develop robust hyperalgesia, unlike clinical postsurgical/dental cohorts where only a subset transitions to persistent orofacial pain. Since chronification reflects evolving multi-level plasticity, discussion of any model or intervention should specify when in the disease course it acts and which trigeminal pathways it engages, acknowledging that current paradigms likely capture only a subset of human mechanisms [90,91].

Another critical translational consideration concerns biological variability, particularly sexual dimorphism in neuroimmune signaling. Evidence from experimental models indicates a striking divergence in the cellular substrates mediating central sensitization between sexes. In males, chronic pain states are predominantly driven by microglial signaling pathways, specifically via TLR4 and purinergic (e.g., P2X4R) activation. Conversely, females frequently bypass these microglial pathways to achieve equivalent nociceptive hypersensitivity, instead relying on adaptive immune mechanisms, most notably T-lymphocyte-mediated cascades. Acknowledging these sex-specific neuroimmune architectures is paramount when translating rodent data from trigeminal pain models into targeted clinical therapeutics [92,93,94].

These findings underscore that neuroimmune mechanisms underlying persistent pain are not uniform across sexes, and that trigeminal pain models limited to young male rodents risk overlooking pathways highly relevant to chronic orofacial pain in females. Incorporating sex as a biological variable is therefore essential for improving the predictive validity of preclinical models and for developing treatments that account for the distinct neuroimmune architectures shaping pain chronification.

To optimize translational fidelity and bridge the gap between preclinical discovery and clinical reality in trigeminal and orofacial pain research, future experimental designs must pivot toward more holistic and longitudinal modeling strategies. First, study timelines must be extended to encompass chronic, longitudinal phases that truly mirror the protracted clinical course of human pathologies such as trigeminal neuralgia, TMJ disorders, and persistent idiopathic facial pain. Second, models must systematically integrate critical strata of biological variability, accounting for sexual dimorphism in neuroimmune profiles, age-dependent changes in glial reactivity, and the presence of affective, anxiety-like comorbidities. Most crucially, preclinical phenotyping should transition away from traditional reflexive hypersensitivity metrics toward a stratified battery of clinically relevant trigeminal readouts.

This multifaceted phenotyping approach should integrate evocative functional assays, such as movement-evoked jaw pain during mastication or blink-reflex metrics to quantify trigeminal brainstem hyperexcitability, alongside validated ongoing pain paradigms like operant conflict tasks to evaluate the cognitive–affective valuation of pain and conditioned place preference to measure the reinforcing value of analgesia. Furthermore, the inclusion of ethologically relevant readouts, such as the facial grimace scale to capture spontaneous, non-evoked pain expressions, is vital to minimize investigator bias and capture the multidimensional sensory, affective, and functional axes of the pain experience. Ultimately, the implementation of these next-generation animal models will yield highly predictive datasets capable of identifying viable, sex-stratified therapeutic targets within the trigeminal system.

The integration of translational neuroscience approaches can substantially strengthen the clinical relevance of glia-focused pain research [41,83,84]. At the cellular level, emerging tools such as single-cell and spatial transcriptomics offer unprecedented resolution for characterizing the molecular heterogeneity of microglia and astrocytes within pain-relevant circuits, potentially revealing previously unrecognized glial subtypes with distinct roles in nociceptive processing. In parallel, metabolic and immunometabolic pathways shape microglial inflammatory phenotypes and influence their capacity to sustain neuroinflammatory signaling, highlighting metabolic modulation as a promising therapeutic strategy [54,95,96].

To enhance translation, particularly for trigeminal/orofacial pain, future models should incorporate longer disease durations, biological variability (sex, age, affective context), and clinically relevant trigeminal outcomes such as movement-evoked jaw pain, blink-reflex metrics, and validated assays of ongoing pain (e.g., operant tasks, conditioned place preference, facial grimace measures).

Despite the insights provided by the included studies, several methodological limitations should be acknowledged. The relatively small number of eligible studies and the presence of moderate-to-high risk of bias in several experiments highlight the need for improved methodological rigor in preclinical pain research. In fact, several studies were classified as having a high risk of bias, mainly due to insufficient reporting of methodological procedures. Therefore, while the overall evidence is consistent, the conclusions should be interpreted with appropriate caution until further well-designed preclinical studies become available. Inadequate reporting of randomization procedures, blinding, and sample size calculations remains common in animal studies and may contribute to overestimates of treatment effects.

An additional limitation of this review is the heterogeneity of the included experimental models. Although all studies investigated mechanisms relevant to trigeminal neuroinflammation and chronic orofacial pain, differences in model induction, behavioral assessments, molecular targets, and biological variables, including sex, limit the direct comparability of the findings and should be considered when extrapolating the results to clinical human conditions. Adoption of rigorous experimental standards and transparent reporting guidelines will be essential to enhance reproducibility and translational potential. Moving forward, the universal adoption and stringent enforcement of standardized frameworks, specifically the ARRIVE guidelines, will be essential to ensure transparent reporting, enhance reproducibility, and maximize the translational fidelity of animal pain models. Finally, it is important to emphasize that the conclusions of this review are based exclusively on evidence derived from experimental animal models, highlighting the need for caution in interpreting all the information summarized.

## 5. Conclusions

This systematic review establishes that the transition from acute injury to chronic orofacial pain depends on a highly coordinated neuroinflammatory cascade that tracks from peripheral tissue to central nociceptive circuits. Rather than relying on purely neuronal pathways, the maintenance of chronic pain is driven by distinct, sequential neuron–glia interactions: peripheral and ganglionic insults disrupt satellite glial cell homeostasis through Kir4.1 downregulation and purinergic P2X shifts, while central sensitization is cemented by early microglial activation, C1q/C3-CR3 synaptic pruning, and delayed, persistent astrocytic Panx1/adenosine feedback loops. This neuroimmune crosstalk extends beyond classical sensory brainstem relays into higher cortical and limbic structures, specifically the medial prefrontal cortex and ventral hippocampus, suggesting a biological framework for the affective, anxio-depressive comorbidities that clinically characterize chronic orofacial pain.

Nevertheless, caution is required when translating these results to human conditions, as most evidence derives from controlled experimental models that may not fully replicate the multifactorial nature and long-term progression of clinical pain disorders. Future research should focus on improving the translational relevance of experimental models, incorporating longer disease courses, sex differences, and behavioral comorbidities. Advances in molecular profiling and neuroimmune imaging may also help clarify the role of glial populations in human chronic orofacial pain and support the development of glia-targeted therapeutic strategies.

Collectively, the evidence supports a sequential neuroimmune model in which peripheral and central glial populations act in a coordinated manner to initiate, amplify, and maintain chronic orofacial pain, providing an integrated conceptual framework for future translational research.

## Figures and Tables

**Table 1:** Characteristics of the included studies.

Reference Type/Country	Animal Model	Pain Model	Observation Period/Timing of Intervention/Primary Behavioral Pain Assessment	Molecular Targets Investigated	Key Findings
[66] EAS/Italy	Male Sprague-Dawley rats	CCI-IoN	26 days after CCI-IoN/Day 26/mechanical allodynia	TRPA1, TLR4/7 signaling in microglia, astrocytes and SGCs	CCI induced neuroinflammation and activation of microglia and SGCs; TRPA1 antagonist reduced cytokine expression and glial activation
[67] EAS/China	Female Sprague-Dawley rats	TMJ osteoarthritis (UAC model)	Baseline, days 14 and 28 after UAC, with post-treatment follow-up/sPLA2-III siRNA or IL-1β antibody injected into the mPFC from day 29, as single or 5-day treatment/orofacial mechanical threshold + hind paw mechanical and thermal tests	Microglial IL-1β signaling in medial prefrontal cortex	Microglial IL-1β increased neuronal PLA2-III expression and promoted orofacial and widespread pain hypersensitivity
[68] EAS/Hungary	Male rats and male Tac4-knockout mice	CFA whisker pad inflammation	1, 3 and 7 days after CFA/CFA injected at baseline (no therapeutic intervention)/Facial mechanonociceptive threshold	Tac4 in neurons and SGCs	Tac4 expression increased during inflammation and contributed to trigeminal pain hypersensitivity
[69] EAS/China	Male and female C57BL/6 mice	CCI-IoN neuropathic pain	Up to 21 days after CION, with main analyses around days 14–21/Pharmacological, optogenetic or chemogenetic modulation, around day 14/Mechanical allodynia + affective behavioral tests	Astrocyte-microglia interaction via adenosine signaling	Astrocyte-derived adenosine and microglial IL-17 signaling in the ventral hippocampus modulated neuropathic pain and anxio-depressive behaviors
[70] EAS/USA	Male and female Panx1-knockout mice	CFA submandibular inflammation	4 weeks after CFA/CFA on day 0; mefloquine administered 2 h before behavioral testing at weeks 1, 3 and 4/Submandibular tactile threshold	Astrocytic Panx1 signaling	Astrocytic Panx1 mediated ATP release and inflammatory signaling and was required for persistent tactile hypersensitivity
[71] EAS/Israel	Male and female Balb/c mice	CFA inflammation and IoN axotomy	1–28 days after; 6–8 days after IoN axotomy/CFA injection or IoN axotomy at baseline; tissue collection according to model/Calcium imaging of SGC ATP responses	Purinergic P2 receptors in SGCs	Inflammation increased ATP sensitivity of SGCs and shifted purinergic signaling from P2Y to P2X receptor dominance
[40] EAS/Italy	Male Sprague-Dawley rats	CFA-induced TMJ inflammation	24 and 72 h after CFA/CFA injected at baseline (no therapeutic intervention)/Orofacial mechanical withdrawal threshold	Microglial purinergic P2Y12 receptor	TMJ inflammation induced microglial activation in trigeminal nociceptive pathways without astrocyte activation
[72] EAS/Canada	Male Sprague-Dawley rats	Mental nerve ligation, LPS or CFA	Up to 28 days after nerve ligation; up to 48 h after LPS or CFA injection/Baseline/Lower lip mechanical allodynia	Microglial and astrocyte activation in trigeminal nuclei	Peripheral nerve injury induced early microglial activation followed by delayed astrocyte activation
[73] EAS/USA	Male Sprague-Dawley rats	Kir4.1 knockdown or CCI-IoN	Up to 13–14 days after Kir4.1 dsRNA injection; CCI-IoN comparison at day 10/injections at baseline; CCI-IoN used as comparator/Orofacial mechanical sensitivity, spontaneous eye closure and operant conflict drinking test	Kir4.1 potassium channel in SGCs	Silencing Kir4.1 in trigeminal SGCs produced pain-like behavior similar to neuropathic injury
[74] EAS/China	Male and female Sprague-Dawley rats	CFA-induced TMJ inflammation	Daily assessment for 3 days after CFA, with molecular analyses on day 3/ANX-005 or minocycline injected into the RVM daily for 3 days/Orofacial mechanical pain threshold	Complement-mediated microglial synaptic pruning (C1q/C3-CR3)	Microglial complement signaling in the RVM promoted synaptic remodeling and mechanical hypersensitivity

Legend: ATP, adenosine triphosphate; Balb/c, bagg albino/c; C57BL/6, dark brown; CCI, chronic constriction injury; CCI-IoN, chronic constriction injury of the infraorbital nerve; CFA, complete Freund’s adjuvant; EAS, experimental animal study; IL, interleukin; Kir4.1, potassium inwardly rectifying channel 4.1; LPS, lipopolysaccharide; mPFC, medial prefrontal cortex Panx1, pannexin-1; PLA2-III, phospholipase A2 group III; RVM, rostral ventromedial medulla; SGCs, satellite glial cells; Tac4, tachykinin precursor 4; TMJ, temporomandibular joint; TLR4/7, toll-like receptor 4 and 7; TRPA1, transient receptor potential ankyrin 1; USA, United States of America.

**Table 2:** Comparative synthesis of the main findings across the included studies.

Aspect	Summary of Findings
Pain models	CCI-IoN, TMJ inflammation, CFA-induced inflammation, mental nerve ligation
Glial cells	Satellite glial cells, microglia, astrocytes
Main anatomical sites	Trigeminal ganglion, trigeminal nucleus caudalis, RVM, hippocampus, PFC
Main molecular pathways	Purinergic signaling, cytokines, TRPA1, complement, Panx1, Kir4.1
Behavioral outcomes	Mechanical allodynia, hyperalgesia, anxiety-/depression-like behaviors
Overall conclusion	All studies supported glial activation associated with chronic orofacial pain

Legend: CCI-IoN, chronic constriction injury of the infraorbital nerve; CFA, complete Freund’s adjuvant; Kir4.1, potassium inwardly rectifying channel 4.1; Panx1, pannexin-1; PFC, prefrontal cortex; RVM, rostral ventromedial medulla; TMJ, temporomandibular joint; TRPA1, transient receptor potential ankyrin 1.

## Data Availability

The original contributions presented in this study are included in the article and Supplementary Material. Further inquiries can be directed to the corresponding author.

## Supplementary Materials

The following supporting information can be downloaded at: https://www.mdpi.com/article/10.3390/pathophysiology33030054/s1, Table S1: PRISMA 2020 checklist.

## Abbreviations

The following abbreviations are used in this manuscript:

ATP	Adenosine triphosphate
BDNF	Brain-derived neurotrophic factor
C1q	Complement component 1q
C3	Complement component 3
CCI	Chronic constriction injury
CCI-IoN	Chronic constriction injury of the infraorbital nerve
CCL2	C-C motif chemokine ligand 2
CD68	Cluster of differentiation 68
CFA	Complete Freund’s adjuvant
CX3CL1	Fractalkine
CXCL1	C-X-C motif chemokine ligand 1
GFAP	Glial fibrillary acidic protein
Iba-1	Ionized calcium-binding adaptor molecule-1
IL	Interleukin
KCC2	Potassium-chloride cotransporter 2
Kir4.1	Potassium inwardly rectifying channel 4
LPS	Lipopolysaccharide
MAPK	Mitogen-activated protein kinase
MeSH	Medical Subject Headings
NMDA	N-methyl-D-aspartate
Panx1	Pannexin-1
PFC	Prefrontal cortex
PLA2-III	Phospholipase A2 group III
PRISMA	Preferred Reporting Items for Systematic Reviews and Meta-Analyses
P2X	Purinergic 2X receptor
P2Y	Purinergic 2Y receptor
ROS	Reactive Oxygen Species
RVM	Rostral ventromedial medulla
SGCs	Satellite glial cells
Tac4	Tachykinin precursor 4
TLR	Toll-like receptor
TMJ	Temporomandibular joint
TNF-α	Tumor necrosis factor alpha
TRPA1	Transient receptor potential ankyrin 1
TSPO	Translocator protein
UAC	Unilateral anterior crossbite
WoS	Web of Science
